# Supplementary material for: Differences between Kazak Cheeses Fermented by Single and Mixed Strains Using Untargeted Metabolomics
Source: Foods. 2022 Mar 26;11(7):966. doi: 10.3390/foods11070966 (PMC8997636; doi:10.3390/foods11070966)
Supplement: Supplementary file 1 [file foods-11-00966-s001.zip › foods-1615606-supplementary.pdf]

Figure S1. Hierarchical cluster analysis of organic acids and derivatives in cheese during ripening. Individual cells corresponded to different cheeses and variables, which are colored red and blue to indicate high and low abundances of these variables, respectively. Abbreviations: M, mixed fermentation cheese; S, single-strain fermentation cheese. 1–40 represent different ripening times (in days).

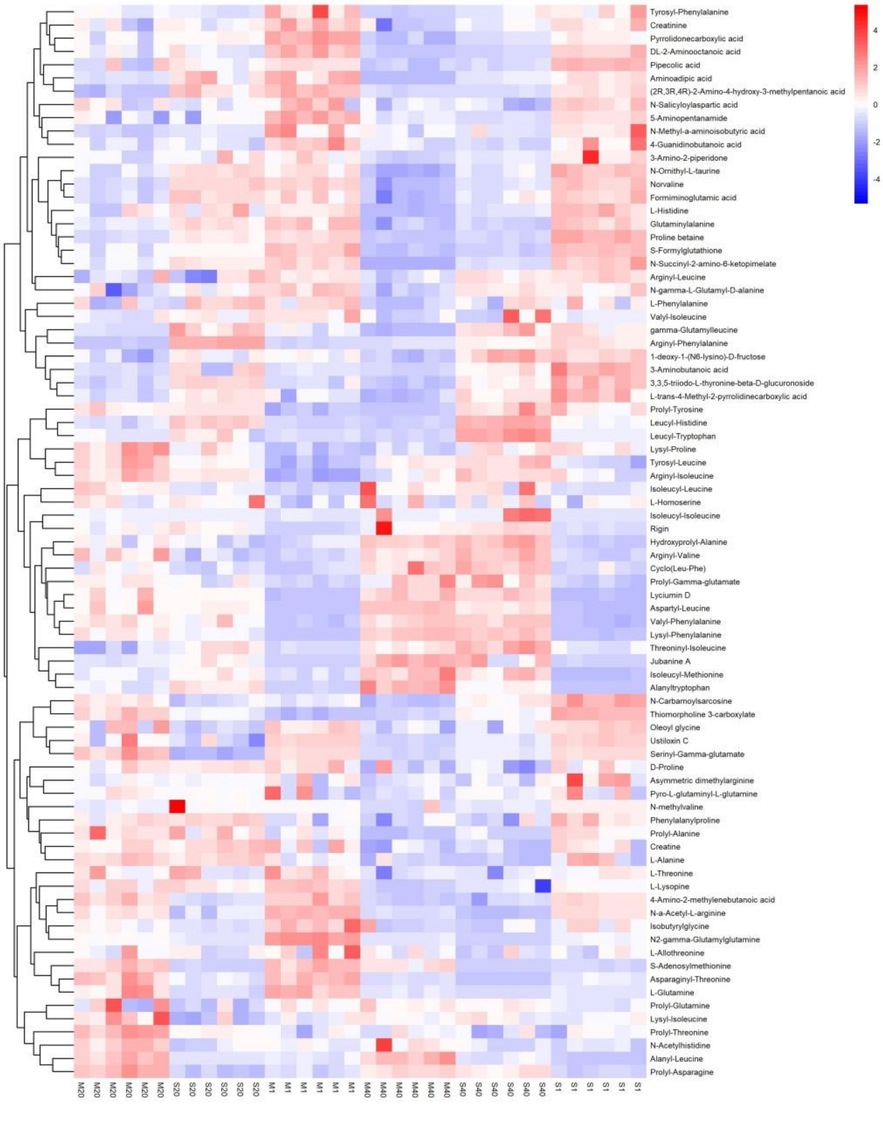

Table S1. Volatile compound contents of cheese (μg/g).

	RI	S1	S10	S20	S30	S40
2-Methyl-, 1-propanol	1092	0	0.120±0.015[a]	0.104±0.007[a]	0.027±0.001[c]	0.058±0.002[b]
1-Butanol	1142	0	0	0	0	0.009±0.000
2-Heptanol	1320	0	0	0.002±0.000	0	0
1-Hexanol, 2-ethyl-	1491	0.017±0.002[c]	0.038±0.005[a]	0.037±0.002[a]	0	0
1-Octanol	1557	0.012±0.001[d]	0.004±0.000[e]	0.024±0.002[a]	0.017±0.001[c]	0.02±0.001[b]
2-Butanol	1025	0	0.005±0.001[a]	0.001±0.000[b]		0
Benzeneethanol	1906	0	0.353±0.025[c]	0.233±0.016[d]	0.581±0.029[b]	1.273±0.051[a]
2-Pentanol	1119	0	0	0	0	0.003±0.000
3-Methyl-, 1-butanol	1209	0.529±0.021[c]	0	0	1.908±0.095[b]	2.935±0.117[a]
Hexanal	1083	0.070±0.009[b]	0	0.122±0.008[a]	0.029±0.001[c]	0.007±0.000[d]
Octanal	1289	0.020±0.003[b]	0.004±0.000[c]	0.045±0.003[a]	0	0
Nonanal	1391	0.042±0.004[b]	0.022±0.001[d]	0.073±0.005[a]	0.014±0.001[e]	0.025±0.001[c]
Pentanal	979	0.001±0.000[d]	0.001±0.000[d]	0.003±0.000[b]	0.002±0.000[c]	0.004±0.000[a]
Acetaldehyde	702	0.001±0.000[c]	0.004±0.000[b]	0.001±0.000[c]	0.004±0.000[b]	0.009±0.000[a]
Benzaldehyde	1520	0.029±0.002[a]	0	0	0.006±0.000[b]	0

Acetic acid	1449	3.447±0.152[a]	3.620±0.183[a]	3.316±0.221[a]	1.818±0.091[b]	1.974±0.079[b]
2-Methyl-, propanoic acid	1570	0.014±0.001[d]	0.051±0.005[a]	0.034±0.002[b]	0.009±0.000[e]	0.022±0.001[c]
Butanoic acid	1625	0.735±0.043[c]	1.427±0.071[a]	1.494±0.075[a]	0.731±0.037[c]	0.943±0.038[b]
3-Methyl-, butanoic acid	1666	0.017±0.002[d]	0.054±0.004[a]	0.042±0.003[b]	0.009±0.000[e]	0.02±0.001[c]
Hexanoic acid	1846	0.642±0.054[c]	1.06±0.101[a]	1.072±0.071[a]	0.795±0.04[b]	1.031±0.041[a]
Heptanoic acid	1950	0.014±0.001[b]	0.011±0.001[c]	0.011±0.001[c]	0	0.042±0.002[a]
Octanoic acid	2060	0.184±0.016[d]	0.248±0.025[c]	0.268±0.018[c]	0.312±0.016[b]	0.435±0.017[a]
Nonanoic acid	2171	0	0.014±0.001[b]	0	0.013±0.001[b]	0.094±0.004[a]
Decanoic acid	2276	0.041±0.003[d]	0.052±0.005[c]	0.051±0.003[c]	0.098±0.005[b]	0.164±0.007[a]
Propanoic acid	1535	0.017±0.002[b]	0.023±0.002[a]	0	0	0
L-Alanine		0	0	0	0.003±0.000	0
2-Heptanone	1182	0.42±0.032[b]	0.471±0.037[b]	0.57±0.038[a]	0.081±0.004[d]	0.108±0.004[c]
2-Butanone, 3-hydroxy-	1284	1.899±0.076[a]	0.865±0.088[b]	0.613±0.041[c]	0.072±0.004[d]	0
2-Nonanone	1390	0.118±0.012[c]	0.163±0.015[a]	0.174±0.012[a]	0.144±0.007[b]	0.168±0.000[a]
2-Undecanone	1698	0.023±0.002[d]	0.029±0.003[c]	0.033±0.002[c]	0.048±0.002[b]	0.063±0.003[a]
2-Pentanone	981	0	0.037±0.002[b]	0.092±0.006[a]	0	0
Ethyl acetate	888	0	0	0	0.11±0.006[b]	0.187±0.008[a]

3-methyl-1-butyl acetate	1122	0	0	0	0.042±0.002[b]	0.11±0.004[a]
Ethyl hexanoate	1233	0	0.048±0.003[c]	0.045±0.003[c]	0.102±0.005[b]	0.409±0.016[a]
ethyl 2-hydroxypropanoate	1349	0	0.368±0.032[b]	0.351±0.023[b]	0.357±0.018[b]	0.664±0.027[a]
Ethyl octanoate	1435	0.012±0.001[c]	0.016±0.002[c]	0	0.074±0.004[b]	0.244±0.010[a]
Decanoic acid, ethyl ester	1638	0	0	0	0	0.044±0.002
2-phenylethyl acetate	1813	0.053±0.004[b]	0.056±0.007[b]	0	0.042±0.002[c]	0.096±0.004[a]
Ethyl butanoate	1035	0	0	0.179±0.012[c]	0.212±0.011[b]	0.443±0.018[a]
Nonanoic acid, ethyl ester	1531	0	0	0	0.015±0.001	0
Hexadecanoic acid, ethyl ester	2251	0	0	0.004±0.000	0	0
Acetic acid, decyl ester	1680	0.047±0.006[b]	0	0.075±0.005[a]	0	0
Acetic acid, methyl ester		0	0.042±0.003[a]	0.027±0.002[b]	0	0
	**RI**	**M1**	**M10**	**M20**	**M30**	**M40**
2-Methyl-, 1-propanol	1092	0	0.137±0.015[b]	0.225±0.009[a]	0.065±0.003[d]	0.086±0.003[c]
1-Butanol	1142	0	0	0	0.003±0.000	0
2-Heptanol	1320	0	0	0.088±0.003	0	0
1-Hexanol, 2-ethyl-	1491	0.017±0.001[c]	0.021±0.002[b]	0.048±0.002[a]	0.010±0.001[d]	0
1-Octanol	1557	0.016±0.001[d]	0.015±0.002[d]	0.030±0.001[b]	0.021±0.001[c]	0.039±0.002[a]

2-Butanol	1025	0.002±0.000[b]	0.002±0.000[b]	0.001±0.000[c]	0.001±0.000[c]	0.005±0.000[a]
Benzeneethanol	1906	0.973±0.065[c]	2.128±0.071[b]	3.289±0.110[a]	2.089±0.070[b]	3.228±0.108[a]
2-Pentanol	1119	0	0	0.003±0.000	0	0
3-Methyl-, 1-butanol	1209	0	0.001±0.000[c]	0	3.231±0.162[b]	4.778±0.191[a]
Hexanal	1083	0.420±0.028[a]	0	0	0.045±0.002[c]	0.112±0.005[b]
Octanal	1289	0.015±0.001[c]	0.011±0.001[d]	0.026±0.001[b]	0.025±0.001[b]	0.073±0.003[a]
Nonanal	1391	0.083±0.006[a]	0.034±0.004[e]	0.049±0.002[d]	0.073±0.004[b]	0.063±0.003[c]
Pentanal	979	0	0.002±0.000[c]	0.009±0.000[b]	0.001±0.000[d]	0.016±0.001[a]
Acetaldehyde	702	0.002±0.000[a]	0	0	0.002±0.000[a]	0.002±0.000[a]
Heptanal	1184	0	0	0.067±0.003[a]	0	0.01±0.000[b]
Decanal	1498	0.004±0.000[a]	0.002±0.000[b]	0	0	0
Acetic acid	1449	0.803±0.054[d]	1.467±0.073[c]	3.116±0.156[a]	1.381±0.069[c]	2.596±0.104[b]
2-Methyl-, propanoic acid	1570	0.058±0.004[c]	0.091±0.008[b]	0.154±0.006[a]	0.027±0.001[e]	0.051±0.002[d]
Butanoic acid	1625	0.314±0.021[e]	0.672±0.060[c]	1.354±0.054[a]	0.545±0.027[d]	1.057±0.042[b]
3-Methyl-, butanoic acid	1666	0	0	0	0.069±0.003	0
Hexanoic acid	1846	0.452±0.030[d]	0.660±0.056[c]	0.948±0.038[b]	0.649±0.032[c]	1.287±0.051[a]
Heptanoic acid	1950	0.005±0.000[c]	0.009±0.001[b]	0	0	0.016±0.001[a]

Octanoic acid	2060	0.14±0.009[d]	0.184±0.017[c]	0.192±0.008[c]	0.255±0.013[b]	0.493±0.02[a]
Nonanoic acid	2171	0.008±0.001[b]	0	0	0.018±0.001[a]	0.019±0.001[a]
Decanoic acid	2276	0.034±0.002[d]	0.044±0.003[c]	0.036±0.001[d]	0.100±0.005[b]	0.199±0.008[a]
Propanoic acid	1535	0	0.028±0.003[b]	0.049±0.002[a]	0	0
L-Alanine		0	0.098±0.010	0	0	0
2-Heptanone	1182	0.139±0.009[c]	0.114±0.011[d]	0.289±0.012[a]	0.052±0.003[e]	0.19±0.008[b]
2-Butanone, 3-hydroxy-	1284	0.159±0.010	0	0	0	0
2-Nonanone	1390	0.072±0.005[e]	0.084±0.006[d]	0.14±0.006[b]	0.102±0.005[c]	0.196±0.008[a]
2-Undecanone	1698	0.018±0.001[e]	0.022±0.002[d]	0.027±0.001[c]	0.046±0.002[b]	0.091±0.004[a]
2-Pentanone	981	0.056±0.004	0	0	0	0
ethyl acetate	888	2.296±0.153[a]	0	0.02±0.001[c]	0.599±0.03[b]	0.647±0.026[b]
3-methyl-1-butyl acetate	1122	0.166±0.011[d]	0.661±0.046[b]	1.999±0.08[a]	0.212±0.011[d]	0.329±0.013[c]
ethyl hexanoate	1233	0	0.156±0.014[d]	0.549±0.022[b]	0.292±0.015[c]	0.951±0.038[a]
ethyl 2-hydroxypropanoate	1349	0.084±0.006[d]	0.423±0.052[c]	1.381±0.055[a]	0.456±0.023[c]	0.964±0.039[b]
ethyl octanoate	1435	0.019±0.001[e]	0.209±0.011[d]	0.355±0.014[c]	0.464±0.023[b]	0.904±0.036[a]
Decanoic acid, ethyl ester	1638	0	0.013±0.001[d]	0.036±0.001[c]	0.088±0.004[b]	0.173±0.007[a]
2-phenylethyl acetate	1813	0.088±0.006[e]	0.343±0.036[d]	0.626±0.025[b]	0.56±0.028[c]	1.25±0.050[a]

ethyl butanoate	1035	0	0.429±0.033^{c}	1.082±0.043^{a}	0.333±0.017^{d}	0.64±0.026^{b}
Nonanoic acid, ethyl ester	1531	0	0	0	0.016±0.001^{a}	0.017±0.001^{a}
Hexadecanoic acid, ethyl ester	2251	0	0	0	0.004±0.000	0
Propanoic acid, 2-methyl-, 3-methylbutyl ester	1189	0	0.008±0.001^{b}	0	0	0.068±0.003^{a}
Heptanoic acid, ethyl ester	1331	0	0	0.018±0.001^{a}	0	0.017±0.001^{a}
ACETIC ACID, METHYL ESTER		0.014±0.001	0	0	0	0

Abbreviations: M, mixed fermentation cheese; S, single-strain fermentation cheese. 1–40 represent different ripening times (in days). Different lowercase letters in each row indicate a significant difference between samples ($P < 0.05$).
